# Novel variant in *NSDHL* gene associated with CHILD syndrome and syndactyly- a case report

**DOI:** 10.1186/s12881-020-01094-y

**Published:** 2020-08-20

**Authors:** D. Hettiarachchi, Hetalkumar Panchal, P. S. Lai, V. H. W. Dissanayake

**Affiliations:** 1 Human Genetics Unit, Faculty of Medicine, University of Colombo, 25, Kynsey Road, Colombo, 08 Sri Lanka; 2 Department of Bioscience, Sardar Patel University, Vallabh Vidyanagar, Gujarat India; 3 Department of Paediatrics, Yong Loo Lin School of Medicine, National University of Singapore, Singapore, Singapore

**Keywords:** CHILD syndrome, X-linked dominant, *NSDHL* gene, Novel variant, Syndactyly

## Abstract

**Background:**

Congenital hemidysplasia with ichthyosiform erythroderma and limb defects also known as CHILD syndrome is an X-linked dominant, male lethal genodermatosis with a prevalence of 1 in 100,000 live births. Mutations in *NSDHL* gene located at Xq28 potentially impair the function of NAD(P) H steroid dehydrogenase-like protein and is responsible for its pathogenesis.

**Case presentation:**

The proband was a 9-month-old twin (T2) girl with a healthy twin sister (T1) of Sri Lankan origin born to non-consanguineous parents. She presented with right sided continuous icthyosiform erythroderma and ipsilateral limb defects and congenital hemidysplasia since birth. Notably the child had ipsilateral hand hypoplasia and syndactyly. There were other visceral abnormalities. We performed whole exome sequencing and found a novel heterozygous variant (*NSDHL*, c.713C > A, p.Thr238Asn).

**Conclusion:**

We report a novel missense variant in the *NSDHL* gene that resides in a highly-conserved region. This variant affects the NAD(P) H steroid dehydrogenase-like protein function via reduction in the number of active sites resulting in the CHILD syndrome phenotype and syndactyly.

## Background

Congenital hemidysplasia with ichthyosiform erythroderma and limb defects also known as CHILD syndrome is an X-linked dominant, male lethal genodermatosis with a prevalence of 1 in 100,000 live births. It is characterized by unilateral inflammatory skin lesions, ipsilateral limb and visceral abnormalities [1, 2]. Thus, so far only 60 cases are reported in literature with twice as more right side involvement than the left. The hallmark skin lesions in this condition is psoriasiform epidermis with hyperkeratosis and parakeratosis along with foam cell infiltration of the papillary dermis. These skin abnormalities are present at birth and are persistent throughout life. However, lesions seem to spare the face and are more commonly seen between the skin folds and creases. Visceral abnormalities may involve the cardiovascular, respiratory, nervous and renal systems. Mutations in *NSDHL* gene located at Xq28 potentially impair the function of NAD(P) H steroid dehydrogenase-like protein which is responsible for its pathogenesis. It is an essential enzyme in the cholesterol biosynthesis pathway [3, 4]. NAD(P) H steroid dehydrogenase-like protein is a C4 demethylase and catalyzes NAD+ dependent oxidative decarboxylation of the C4 methyl groups of 4α-carboxysterols [3, 4]. The latter is involved in post-squalene cholesterol biosynthesis and is also a regulator of the Epidermal Growth Factor Receptor (EGFR) trafficking pathways. There is a dual localization of this protein, within the membranes of endoplasmic reticulum and on the surface of lipid droplets. Dysregulation of this pathway has also shown to cause metabolic derangements such as hypercholesterolemia, certain cancers, cardiovascular diseases and neuropathies [5–7]. Here we report the first Sri Lankan patient with CHILD syndrome due to a novel heterozygous variant (*NSDHL*, c.713C > A, p.Thr238Asn).

## Case presentation

**Fig. 1:**
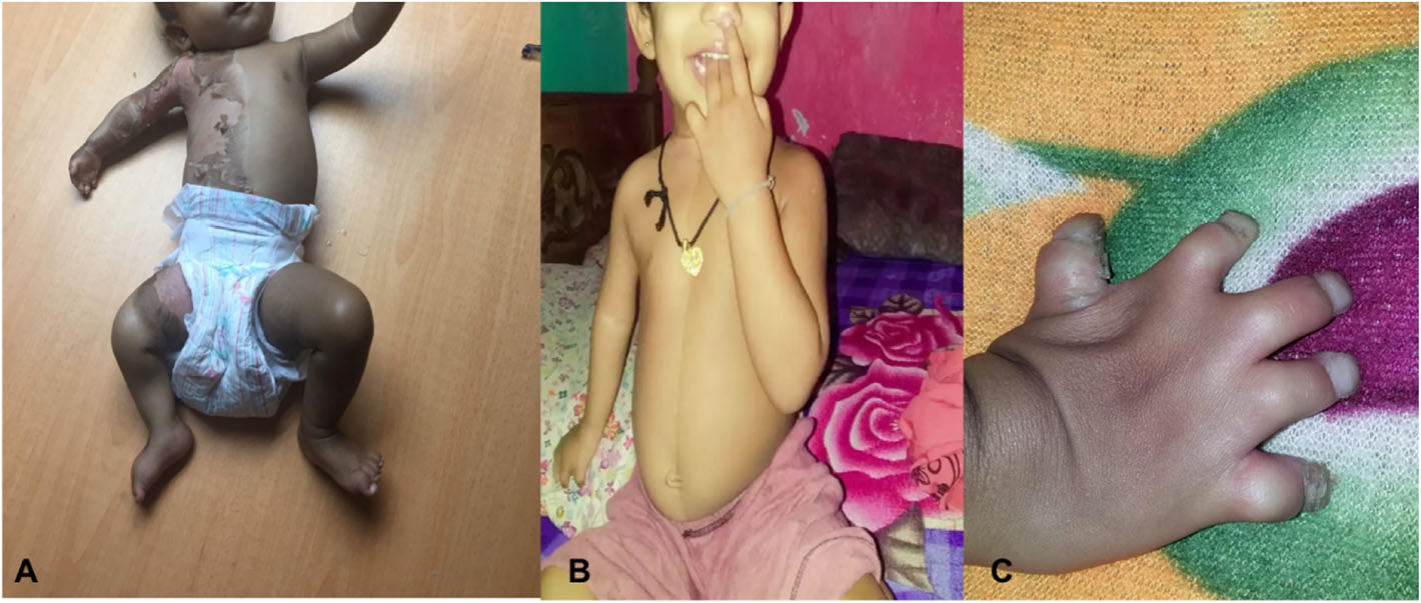
**a**–Proband at 9 months with ipsilateral ichthyosiform erythroderma and Hypoplasia of arm and hand. **b**- Proband after 1 year, following treatment. Images showing remarkable improvement in the skin lesions. **c**- Simple syndactyly of right hand with nail changes

The proband was a 9-month-old twin (T2) girl of Sri Lankan origin born to non-consanguineous parents and her twin sister (T1) was healthy. She presented with congenital hemidysplasia of the right side, ipsilateral limb defects and icthyosiform erythroderma which was continuous from her neck to mid-thigh including the upper arm. There were other visceral abnormalities such as; an absent right (ipsilateral) kidney and.

asymmetrical ventriculomegaly (L > R) in the brain scan. However, we noticed that the child had hypoplastic hands with syndactyly a feature which was not reported in literature previously (Table 1). We performed whole exome sequencing and found a novel heterozygous variant (*NSDHL*, c.713C > A, p.Thr238Asn). She was diagnosed with Congenital hemidysplasia with ichthyosiform erythroderma and limb defects (CHILD) syndrome, in-silico analysis predicted this as a pathogenic variant. She had undergone traditional Sri Lankan herbal medicine in the form of topical and oral preparations for the skin lesions for less than a year. Which has shown significant improvement (Fig. 1a,b,c). However, the exact details of the preparations used was not disclosed.

**Table 1:** Comparison of clinical data of missense variants in *NSDHL* gene

Clinical features	Other missense variants in *NSDHL* gene [8]	Proband
Ipsilateral CHILD nevus	both diffuse and wide spread involvement of trunk, thigh and groin area	continuous involvement of neck trunk, upper arm, hand including web spaces groin, upper thigh and right foot.
Laterality	ipsilateral, contralateral lesions and bilateral lesions are also reported [9]	ipsilateral lesions confined to the right side with strict midline demarcation
Ipsilateral extracutaneous defects	hypoplasia of arm, leg, foot, pelvis and skull	hypoplasia of upper limb with simple syndactyly of all fingers with nail changes of the right hand. Aplastic femoral head, length of right leg shorter than the left leg and normal foot.
Visceral abnormalities	ipsilateral visceral abnormalities	absent right (ipsilateral) kidney. ultra sound scan of brain- asymmetrical ventriculomegaly (L > R)
Hearing defects and vocal cord involvement	observed in some cases	not observed

### In-silico analysis

The initial analysis was done using the National Center for Biotechnology Information (NCBI) database, mRNA sequence of NAD(P) H steroid dehydrogenase-like protein (NSDHL)(XM_011531178.2; https://www.ncbi.nlm.nih.gov/nuccore/XM_011531178.2) was retrieved in FASTA format from NCBI with NCBI Reference Sequence ID: NP_057006.1 having 373 amino acids. Observed variation in proband was done manually in the mRNA sequence and taken for further analysis as c.713C > A, p.Thr238Asn.

UniProt database for protein sequences and functional information revealed the Protein sequence of NAD(P) H steroid dehydrogenase-like protein (NSDHL) which was retrieved in FASTA format from UniProtKB repository having unique ID Q15738 (NSDHL_HUMAN). This protein sequence was taken as a reference sequence for comparisons of variant protein due to variations in DNA sequence and in protein sequence. There were also some previously reported variations in the Uniprot database and they were p.Alar105Val, p.Ala182Pro, p.Gly205Ser [10]. I-Tasser an online bioinformatics server for the prediction of protein structure and function (https://zhanglab.ccmb.med.umich.edu/I-TASSER/) was then used to form the 3D construct [11]. I-TASSER detects templates from PDB and constructs full length protein models by replica exchange Monte-Carlo simulations. Sequences of normal and mutated NSDHL protein were submitted to I-TASSER for 3D structure prediction and predicted protein models were downloaded and analyzed. Followed by automated homology modeling using the Swiss-PdbViewer [12]. Both the proteins were then compared with reference to active sites present in them. Stereochemical quality of the protein structure was then analyzed using PROCHECK. Thus, a Ramachandran plot was obtained which showed energetically allowed regions for backbone dihedral angles ψ against φ of amino acid residues in protein structure [11].

In silico analysis using nine computational prediction tools namely DANN, DEOGEN2, FATHMM-MKL, M-CAP, MVP, MutationAssessor, MutationTaster, REVEL and SIFT [13, 14] returned results of likely pathogenic effects due to NSDHL, c.713C > A, p.Thr238Asn This variant is also not found in all known population databases or in our in-house Sri Lankan population database (Supplementary file).

**Fig. 2:**
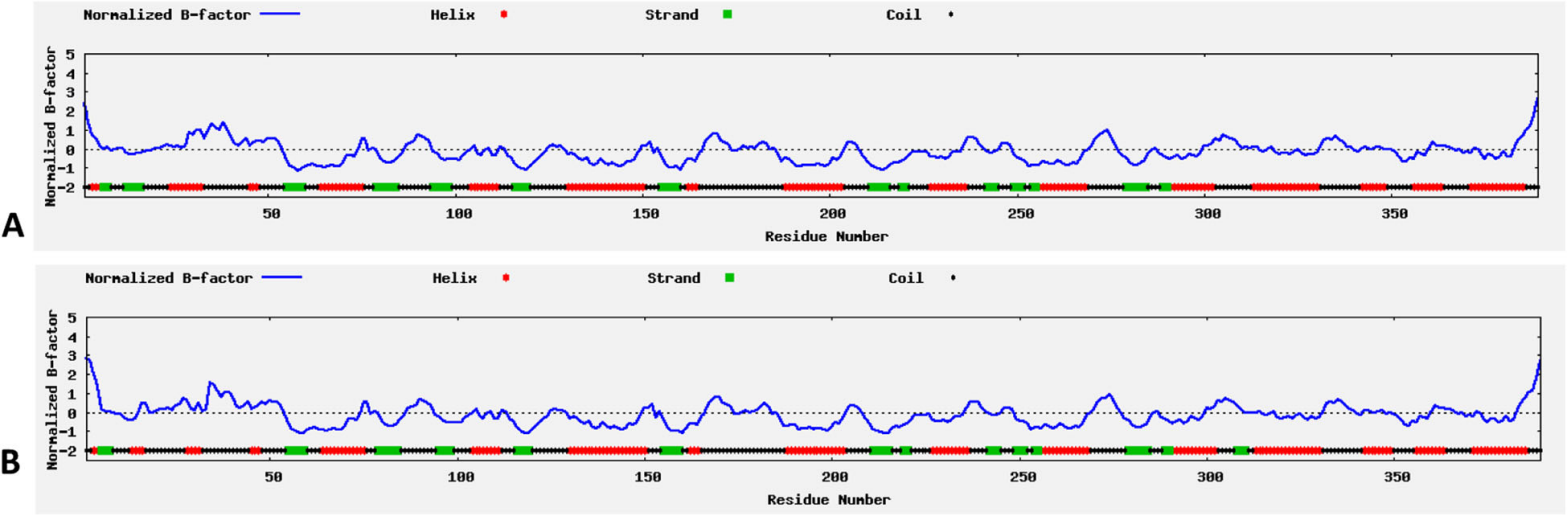
**a-** Distribution and B factor values of alpha helices and beta sheets in wild type protein,**b** - In mutant protein both show similar distribution patterns

**Fig. 3:**
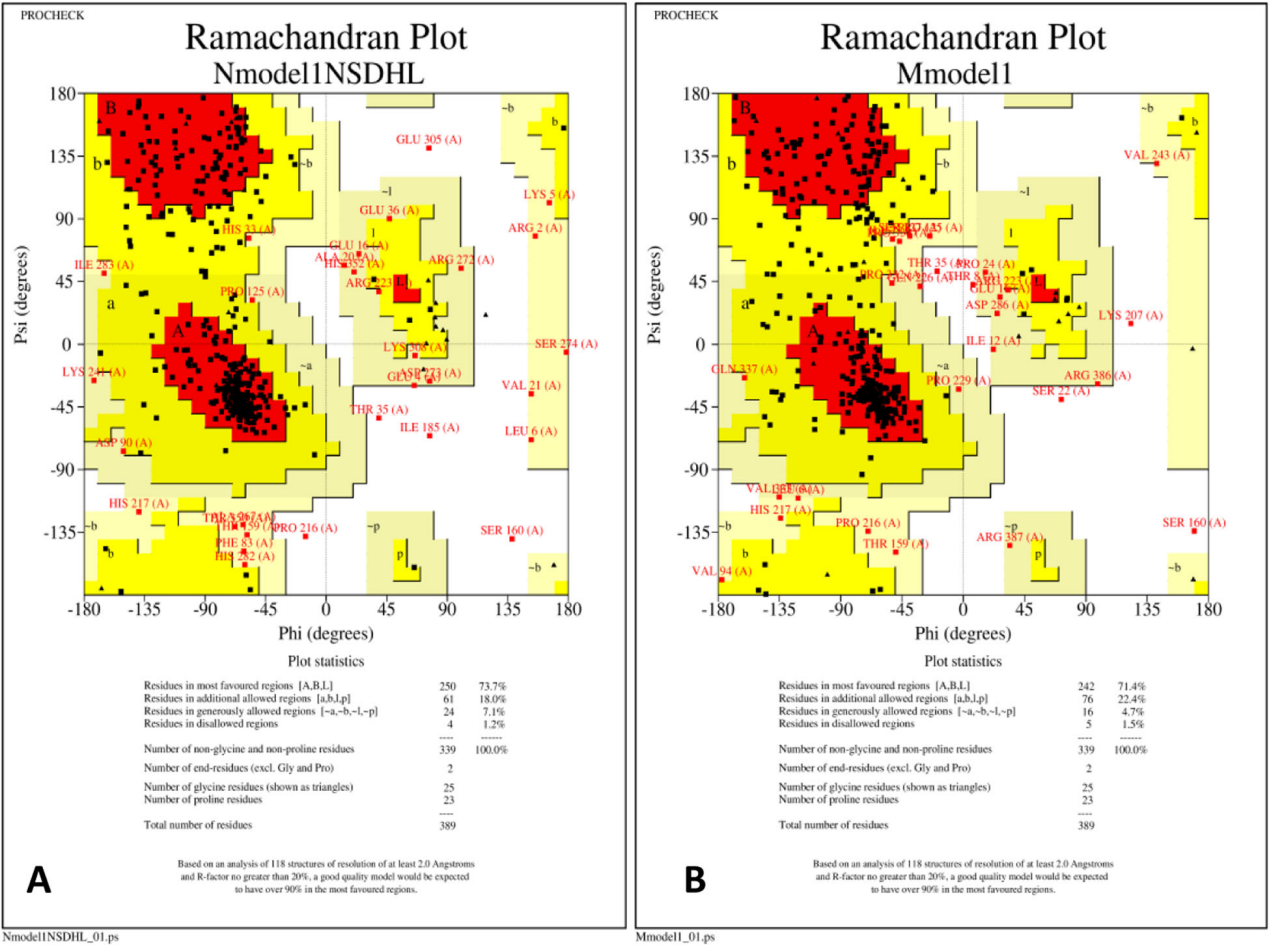
**a**- Ramachandran plot of wild type NHDL protein; The wild type protein have residues in the following region of the plot, 250 (73.7%) in the preferred regions such as A, B, L; 61 (18.0%) in a,b,l,p regions; 24 (7.1%) in regions ~a,~b,~l,~p and only 4 (1.2%) in unfavorable regions. **b**- Ramachandran plot of mutant NHDL protein; In the mutant proteins we observed 242 (71.4%) in A, B, L; 76 (22.4%) in a,b,l,p regions; 16 (4.7%) in regions ~a,~b,~l,~p and 5 (1.5%)) in unfavorable regions

**Fig. 4:**
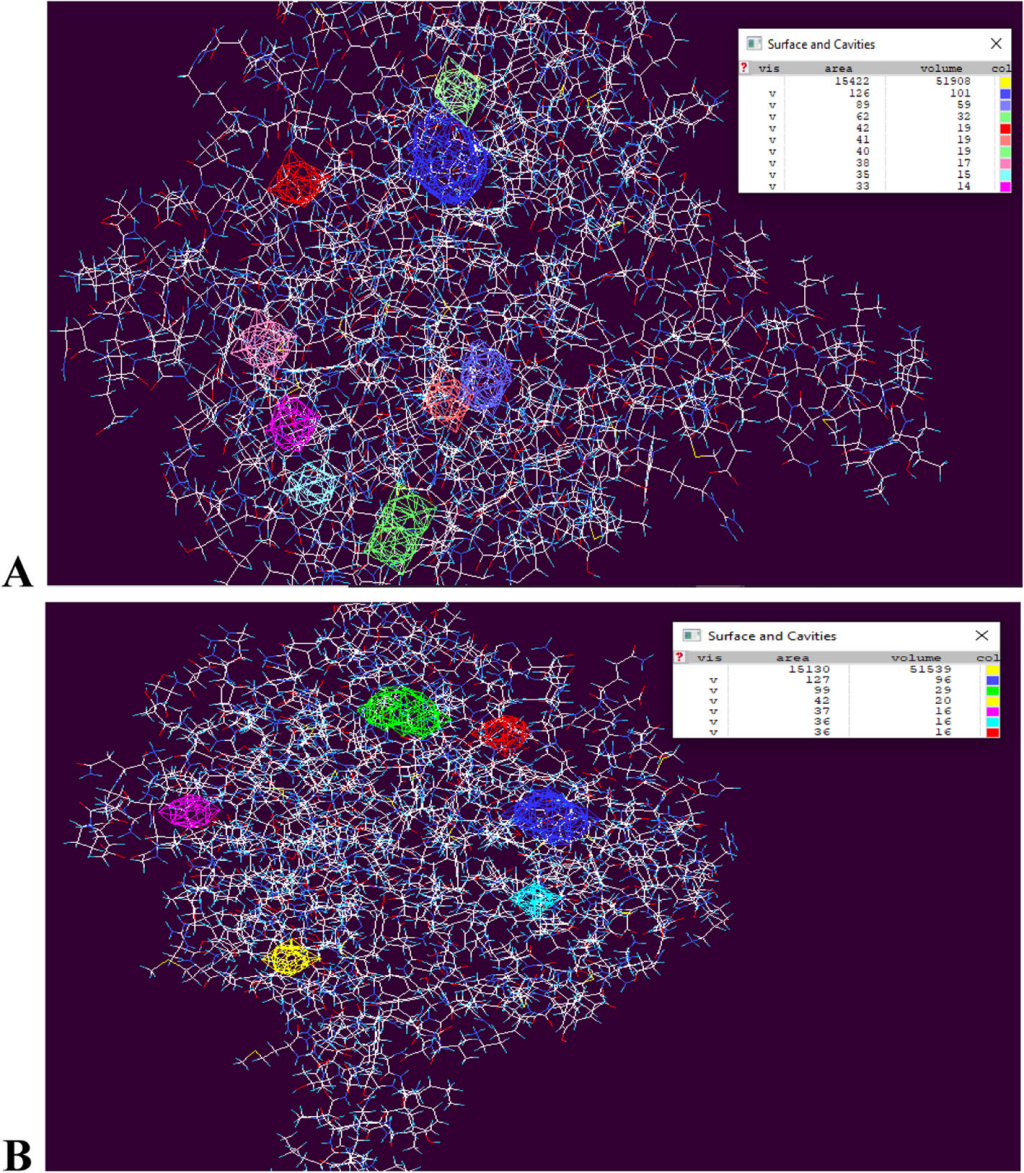
**a:** Wild type NSDHL protein structure with nine active sites, **b** Mutant NSDHL protein structure with only six active sites

Further, after modeling normal and the NSDHL proteins of the proband we found that there is a difference in the secondary and tertiary structure. Number of alpha helices and beta sheets were different in both proteins. There is a difference in the starting and the ending positions of the secondary structures and also in the spatial arrangement of alpha helices and beta sheets in the 3D folding. The accuracy of secondary structures was also perfect and supported by value of normalized B-factor. Normalized B-factor for a target protein is defined as z-score-based normalization of the raw B-factor values. In our predicted model majority of secondary structures had less than 0 values indicating its stability. The estimated normalized B-factor is shown in Fig. 2a & b. We compared the Ramachandran plots of the wild-type protein and mutated protein (Fig. 3a & b). The wild type protein had residues in the following region of the plot, 250 (73.7%) in the preferred regions such as A, B, L; 61 (18.0%) in a,b,l,p regions; 24 (7.1%) in regions ~a,~b,~l,~p and only 4 (1.2%) in unfavorable regions. In the mutant proteins we observed 242 (71.4%) in A, B, L; 76 (22.4%) in a,b,l,p regions; 16 (4.7%) in regions ~a,~b,~l,~p and 5 (1.5%)) in unfavorable regions. We also observed that the variant found in the proband reduced the number of active sites in the resultant protein. Normal protein has 9 active sites whereas variant protein has only 6 active sites (Fig. 4a & b). We also analyzed the differences in the protein surface to determine the type of interactions. The difference in the cavities between the wild type and the mutant protein is described in Table 2A and B respectively. As per our analysis we found less number of cavities/ pockets in the mutated protein. Thus, reducing its ligand binding potential. The exact pathways that these ligands facilitate is yet to be determined. However, this leads to a reduction in the number of interacting molecules. Hence, we speculate that there is a significant reduction in the protein function.

**Table 2:** Detailed description of cavities in the wild type protein

Sr. No.	Area cavity	Volume of cavity	Amino acids present in cavity
**Normal Protein cavities details**			
1	126	101	ILE12, MET17, GLY61, SER62, GLY63, GLN67, HIS68, ASP90, ASN91, ARG223, PRO225.
2	89	59	ILE165, PHE166, GLU167, GLY168, ASP170, LYS172, ASN173, GLY174, LEU178, THR350, PHE351, HIS352, TYR353, TYR354.
3	62	32	GLU196, VAL199, LEU200, ASN203, THR211, ALA213, GLY278.
4	42	19	THR32, HIS33, PHE255, GLU257, ASN258, LEU368, VAL369.
5	41	19	SER161, ALA162, ILE165, ARG215, HIS282, TYR254.
6	40	19	PHE15, GLY221, PRO222, ARG223, ASP224, PRO229, THR378, SER381, LEU385.
7	38	17	LEU73, ARG76, TYR78, PHE118, ILE264, ALA267, GLU268
8	35	15	LYS155, LEU156, ILE157, LEU210, THR211, THR212, LEY276.
9	33	14	THR116, VAL117, PHE118, LYS155, ILE157, ALA267.
**Mutated Protein cavities details**			
1	127	96	PHE13, MET17, SER62, GLY63, PHE64, GLN67, PHE89, ASP90, ASN91, ARG223.
2	99	29	LYS40, ASN42, GLU257, ASN258, HIS261, GLY262, LEU265, TYR365, GLN366, LEU368.
3	42	20	PHE166, GLU167, VAL169, LEU347, THR350.
4	37	16	VAL199, LEU200, ASN203, ASP204, THR211, GLY278.
5	36	16	LEU231, ILE232, PHE294, LEU295, ILE298.
6	36	16	VAL70, LEU74, VAL80, VAL82, ASN91, PRO92, GLN93, VAL94.

## Discussion & conclusion

CHILD syndrome is a X linked dominant condition with multisystem involvement showing a unique lateralization pattern and a strict midline demarcation of an inflammatory epidermal nevus. NAD(P) H steroid dehydrogenase-like protein (Nsdhl), which is responsible for this condition is a C3 sterol dehydrogenase enzyme involved in the conversion of lanosterol into cholesterol. Hence, the CHILD phenotype could result from the lack of cholesterol or other sterols downstream due to the block in its biosynthesis or due to the accumulation of products upstream to this pathway. In cell lines where a missense variant in the *NSDHL* gene was introduced, it was found that there was a significant interruption to this pathway as compared to wild-type NSDHL cell lines [8, 15]. In keeping with the current literature, molecular genetic analysis in our patient strongly predicted that CHILD syndrome was caused by a novel missense variant in *NSDHL* it was also found that the variant was located in a highly-conserved site in the NAD(P) H steroid dehydrogenase-like protein across other species as well (Table 3). Syndactyly was a unique feature we observed in this child and it could be due to cholesterol metabolism pathways acting downstream of cytochrome P450 oxidoreductase enzyme and its involvement in skeletal development [16].

**Table 3:** Conservation of protein domain across species corresponding to p.Thr238Asn

Species	Sequence
Proband	IGNGKNLVDF**A**FVENV
*Homo sapiens* Human	IGNGKNLVDF**T**FVENV
*Mus musculus* House mouse	IGNGENLVDF**T**FVENV
*Rattus norvegicus* Norway rat	IGNGKNLVDF**T**FVENV
*Danio rerio* Zebrafish	IGDGSNLVDF**T**YVENV
*Bos Taurus* Cattle	IGNGKNLVDF**T**FVENV
*Canis lupus familiaris* Dog	IGNGENLVDF**T**FVENV
*Gallus gallus* Chicken	IGDGKNLVDF**T**YVENV
*Macaca mulatta* Rhesus monkey	IGNGKNLVDF**T**FVENV
*Xenopus tropicalis* Tropical clawed frog	IGNGKNLVDF**T**YVENV
*Pan troglodytes* Chimpanzee	IGNGKNLVDF**T**FVENV

By analyzing the Ramachandran plot of the two proteins we observed that its structural stability of the protein was unaffected. However, through *in-silico* protein modeling studies we also observed that the wild type protein had 9 active sites whereas the mutated protein in our patient had only 6. Hence, we suspect the 3 missing active sites are crucial for its enzymatic function. All computational and predictive analyses thus indicate *NSDHL*, c.713C > A, p.Thr238Asn as a null variant resulting in loss of function of *NSDHL* gene that is already known to lead to CHILD syndrome. This report contributes to the growing list of missense variants that have been associated with CHILD syndrome [8, 9] and facilitates discovery of more patients with similar variant.

We report a novel missense variant in the *NSDHL* gene residing in a highly-conserved region which affects the NAD(P) H steroid dehydrogenase-like protein function via reduction in the number of active sites. This variant expands the phenotype and genotype data suspected to be associated with CHILD syndrome.

## Supplementary information

**Additional file 1: Table 1.** Pathogenicity prediction and conservation scores for NSDHL c.713C>A p.Thr238Asn. **Table 2.** Frequency of NSDHL c.713C>A p.Thr238Asn in global population databases

## Data Availability

The datasets generated and analysed during the current study are available in GenBank accession number: MT747166, link- https://www.ncbi.nlm.nih.gov/nuccore/MT747166

## Abbreviations

SPDBV: Swiss-PdbViewer
I-TASSER: Iterative Threading ASSEmbly Refinement
NCBI: National Center for Biotechnology Information
EGFR: Epidermal Growth Factor Receptor
