# Supplementary material for: Novel variant in NSDHL gene associated with CHILD syndrome and syndactyly- a case report
Source: BMC Med Genet. 2020 Aug 20;21:164. doi: 10.1186/s12881-020-01094-y (PMC7439548; doi:10.1186/s12881-020-01094-y)
Supplement: Supplementary file 1 — Additional file 1: Table 1. Pathogenicity prediction and conservation scores for NSDHL c.713C>A p.Thr238Asn. Table 2. Frequency of NSDHL c.713C>A p.Thr238Asn in global population databases [file 12881_2020_1094_MOESM1_ESM.docx]

Table 1: Pathogenicity prediction and conservation scores for NSDHL c.713C>A p.Thr238Asn

| Pathogenicity Scores | | |
| --- | --- | --- |
| Tool | Score | Prediction |
| DANN | 0.9937 | Pathogenic |
| MutationTaster | 1 | Disease causing |
| Mutation assessor | 3.365 | Medium |
| FATHMM-MKL | 0.9951 | Damaging |
| DEOGEN2 | 0.9437, 0.7895 | Damaging |
| SIFT | 0.002 | Damaging |
| MVP | 0.9203 | Pathogenic |
| REVEL | 0.8 | Pathogenic |
| M-CAP | 0.161 | Possibly Pathogenic |

| Conservation Scores | |
| --- | --- |
| Tool | Score |
| GERP RS | 5.2899 |
| PhyloP17way | 0.5989 |
| PhyloP30way | 1.026 |
| PhyloP100way | 5.952 |
| PhastCons17way | 0.9559 |
| PhastCons30way | 1 |
| PhastCons100way | 1 |
| MPC | 0.8523 |
| bstatistic | 579 |

Table 2 Frequency of NSDHL c.713C>A p.Thr238Asn in global population databases

| Population frequencies | |
| --- | --- |
| GnomAD Exomes | Variant not found in GnomAD exomes (good GnomAD exomes coverage = 73.0). |
| GnomAD Genomes | Variant not found in GnomAD genomes (good GnomAD genomes coverage = 22.8). |
| ESP | Not found |
| 1000g | Not found |
